# UK paediatric clinical trial protocols: A review of guidance for participant management and care in the event of premature termination

**DOI:** 10.1177/17407745241296864

**Published:** 2024-11-19

**Authors:** Helen Pluess-Hall, Paula Smith, Julie Menzies

**Affiliations:** 1 University of Bath, Bath, UK; 2 University Hospitals Bristol and Weston NHS Foundation Trust, Bristol, UK; 3 University of Birmingham, Birmingham, UK

**Keywords:** Clinical trial, protocol, premature termination, clinical research, early termination, research participation, children, paediatric

## Abstract

**Background/Aims:**

Clinical trials provide an opportunity to identify new treatments and can offer patients access to treatments otherwise unavailable. However, approximately 10% of paediatric clinical trials discontinue before the trial has completed. If this premature termination is because the trial treatment(s) being investigated are identified to be ineffective or unsafe, it results in the abrupt discontinuation of the investigational medicinal product for participants. For some participants, there may not be other treatment options to pursue at the trial-end. Trials prematurely terminating can be a distressing experience for all involved and currently there is little published evidence about the guidance provided to healthcare professionals in the event of premature trial termination. The study protocol is the source of guidance for healthcare professionals delivering clinical research, detailing how to conduct all aspects of the trial. The aim was to quantify the proportion of clinical trial protocols that included premature trial termination and subsequently those that provided instructions related to participant management and care. In addition, to analyse the context in which premature termination was included and the detail of any instructions for participant management and care.

**Methods:**

The ClinicalTrials.gov database was searched by a single reviewer for UK interventional drug trials enrolling children with an available study protocol. Protocols were searched to assess if the risk of premature trial termination was identified, the context for premature termination being included, if information was provided to support the management and care of participants should this situation occur and the detail of those instructions. Data were summarised descriptively.

**Results:**

Of 245 clinical trial protocols, 235 (95.9%) identified the possibility of premature trial termination, the majority within the context of the sponsor asserting their right to terminate the trial (82.7%, 115/235) and providing reasons why the trial could be stopped (65.5%, 91/235). Forty-two percent (98/235) provided guidance for participant management and care, most commonly to contact/inform the participant (45.9%, 45/98). Directions varied in the quantity and level of detail.

**Conclusions:**

This review of UK clinical trial protocol highlights that information surrounding premature termination is lacking, with only 42% providing guidance on the care of trial participants. While this ensures regulatory compliance, it fails to consider the challenge for healthcare professionals in managing participants on-going care or the duty of care owed to participants. Further research is required to understand if additional documents are being used in practice, and if these meet the needs of healthcare professionals in supporting research participants and families during premature trial termination.

## Background

Clinical trials allow new treatments to be developed, participation may provide access to treatments otherwise unavailable and potentially a direct clinical benefit.^1,2^ The expectation when enrolling clinical trial participants is that they will follow a pathway clearly described within the protocol and associated documents. However premature termination, the trial ending earlier than originally planned, occurs in approximately 10% of paediatric trials^3^ and while there is no overarching figure for adult trials, it has been shown to occur in 23.1% of adult oncology trials.^4^ This is predominantly due to insufficient recruitment;^5^ however, it also results from sponsor decision-making and withdrawal of support for the trial such as favourable opinions, in the case of the United Kingdom, from the Research Ethics Committee or Medicines and Healthcare products Regulatory Agency.

Trials should be prematurely terminated if the trial treatment is clearly (1) better than the control, (2) worse than the control, and (3) not going to be shown to be better than the control,^6^ summarised as stopping for efficacy, inferiority, and futility.^7^ A paucity of literature and lack of standardised reporting makes quantifying the overall proportion of trials prematurely terminating and for which reasons difficult. Studies have reported interventional trials terminated because of data from the trial (efficacy or toxicity; 21%),^5^ informative termination which included changes to standard of care and safety or efficacy findings (12.5%),^8^ benefit/harm (6%),^9^ futility (5%),^9^ and unknown reasons (5%–9.6%).^5,8,9^

How premature trial termination impacts participant’s treatment can depend on the termination reason. When the trial treatment is shown to be of benefit, continuing to randomise participants to alternatives would be unethical. Participants may all be offered the effective trial treatment while the sponsor applies for wider usage approval. Where there is inferiority or futility, the treatment is abruptly stopped. For participants who have completed their trial involvement or have finished receiving the investigational treatment and remain in follow-up, there will be no treatment impact, but there may be a need for additional follow-up.

When a clinical trial is prematurely terminated, it is the healthcare professionals delivering the research that are required to manage the participant, their family, and the situation. The protocol is the key clinical trial document, describing the scientific justification, objectives, methodology, statistical considerations, and organisation of the trial.^10^ It should include all the processes and procedures required to be undertaken and is where healthcare professionals seek trial-specific information.

The review aimed to quantify the proportion of paediatric clinical trial protocols that included premature trial termination and provided instructions for participant management and care. In addition, to analyse the context in which the premature termination was included and the detail of any instructions for participant management and care.

## Methods

### Search and eligibility

The publicly accessible ClinicalTrials.gov database was selected as an established database cataloguing clinical trials being conducted globally. Its use is recommended by the UK Health Research Authority,^11^ mandated by the US Food and Drug Administration^12^ and accepted by the International Committee of Medical Journal Editors as a registry location that satisfies the conditions for manuscript publication.^13^ Therefore, it is well-used by researchers internationally and one of the largest databases of clinical trials.^14^ The database was searched (10/1/2022) applying the filters United Kingdom, intervention of drug, child (birth to 17 years old) and with an available study protocol. No date restrictions were applied. The trial details were screened and all eligible trials were included in the review.

### Inclusion criteria

Clinical Trials were included if they were being conducted within the United Kingdom, with paediatric participants (0–17 years old), the intervention under investigation was a drug, and the protocol was available in full through the ClinicalTrials.gov database.

### Exclusion criteria

The following clinical trials were excluded: Participants were exclusively adults (defined as over 18); maternal studies with the pregnant women receiving the intervention but the newborns included as participants for follow-up; exclusively conducted outside of the United Kingdom, to capture trials within the National Health Service (NHS) setting; without an available protocol on the database; device studies as the category encompasses devices at the patient level, for example, insulin delivery pumps, along with devices at service level, for example, magnetic resonance imaging (MRI) scanners. A premature termination of a trial investigating a device at the service level would not have an immediate consequence for the participant or require the patient to be managed by their healthcare professional in the same way as an investigational medicinal product study.

### Review

**Figure 1.**
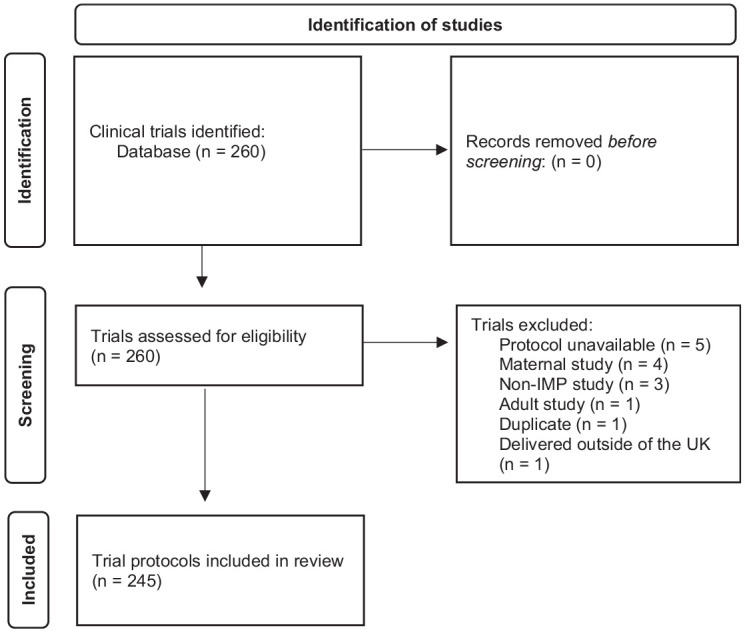
Flow diagram of the identification of clinical trial protocols for this review.

As a single database review using a systematic approach this is reported using the PRISMA (Preferred Reporting Items for Systematic Reviews and Meta-Analyses) guidance.^15^ As summarised in Figure 1, the search identified 260 clinical trials of which 15 were excluded, resulting in 245 protocols being included in the review. The clinical trials included recruited their first patients between January 2007 and September 2021. The review was conducted on the latest protocol version for each clinical trial, the date range of which was March 2013 to May 2021. A comprehensive review was conducted, by a single reviewer, to determine if and in what context the protocol identified the potential for the clinical trial to be prematurely terminated and if any details for how to care for trial participants upon premature trial termination were provided. Protocols were reviewed using keyword searches, examining the schedule of events, study visit details, and any other potentially relevant section as determined by the contents.

### Analysis

Descriptive statistics were used to measure frequency and answer to the review questions. Any information protocols included about premature termination was captured and grouped to allow the context and relevance for healthcare professionals to be reviewed.

## Results

### Identifying the potential for premature termination

Of the 245 eligible protocols, 95.9% (235/245) stated the possibility of premature trial termination, 58% (137/235) of those contained no information about how to manage and care for participants in the event of the trial terminating despite premature termination being noted in multiple sections (see Table 1). Of those protocols which didn’t include information on participant management and care in relation to premature trial termination, the most common context was the sponsor asserting their right to terminate the trial (83.9%, 115/137) and providing reasons why the trial could be stopped (66.4%, 91/137; see Supplementary Table 1). About 3.6% (5/235) of protocols that did not include guidance for participant management noted that if the trial was to be prematurely terminated the sponsor would provide information. In addition, 5.1% (7/235) stated discontinuation procedures would be arranged.

**Table 1.** Context in which protocols not providing guidance for participant management identified premature termination.

Context in which protocol identified premature termination	Number of protocols
Asserting right to terminate the study	115
Reasons why the trial may be terminated	91
Investigator or sponsor to notify regulatory bodies	56
Sponsor will discuss with or notify the investigator	40
Sponsor and/or investigator will ensure adequate consideration is given to the protection of subject’s interests	11
Stopping rules/criteria	7
Sponsor and investigator will arrange discontinuation procedures	5
Sponsor will provide written instructions for termination	4
Designated public website will be updated	4
DSMB/DMC will provide recommendations to sponsor including study termination	3
Schedule for termination will be instituted	2
Procedures will be arranged after sponsor and investigator consultation	2
Investigator will be reimbursed for participants treated	2
ESC will decide to continue or terminate based on DSMC recommendations	1
Reasons that may alter study duration	1
Study-specific procedure for early termination will be provided	1

DSMB: Data and Safety Monitoring Board; DMC: Data Monitoring Committee; DSMC: Data and Safety Monitoring Committee; ESC: Executive Steering Committee.

### Participant care

Of the protocols identifying premature termination (95.9%, 235/245), 42% (98/235) provided guidance for participant management and care, with variation in the quantity of instructions and level of detail. The most included instruction was for the investigator to contact/inform the participants (45.9%, 45/98; see Table 2). About 7.2% (17/235) provided conflicting information, listing trial termination as a reason for participants discontinuing the investigational medicinal product while describing the follow-up needed for participants who discontinue the investigational medicinal product only in the context of the trial continuing.

**Table 2.** Guidance provided within the protocol for healthcare professionals on the management and care of participants if the trial prematurely terminates.

Instructions for healthcare professionals	Number of protocols
Inform/contact participants	45
Conduct an identified study visit	36
Ensure/provide relevant/appropriate medical treatment and follow up	20
See the participant as soon as possible	17
Follow any additional procedures the investigator is informed of	17
Undertake visits/follow-up for discontinuing participants	17
Instruct participants to discontinue IMP	3
Withdraw patients from the study	2
Undertake additional visits beyond those in the protocol, if necessary, to ensure adequate safety monitoring	2
Implement the termination in a time frame compatible with patient wellbeing	1
Treat the participant as per standard of care after stopping IMP	1

IMP: Investigational Medicinal Product.

## Discussion

About 95.9% of clinical trial protocols for interventional drug trials enrolling paediatric participants within the United Kingdom include the possibility of the trial terminating prematurely. This is mainly in the context of the sponsor asserting the right to terminate (83.9%) and the reasons for stopping (66.4%). Only 42% of protocols provided guidance for participant management and care, most commonly the instruction for the investigator to contact/inform the participants (45.9%).

All clinical trials have the potential to prematurely terminate and immediately end the participants research journey. Healthcare professionals delivering clinical research will have undertaken research training such as Good Clinical Practice,^16^ which affirms that clinical trial protocols will be clear and detailed. This creates an assumption that the protocol will contain all the information required to manage and care for participants; however, this review highlights that in the event of premature termination, this is not the case. Identifying the possibility of premature termination, asserting the sponsor’s right to terminate the trial and the reasons why it might be stopped demonstrates a compliance with the regulatory guidance^17^ and Standard Protocol Items: Recommendations for Interventional Trials (SPIRIT) reporting guidance,^18^ which aims to improve the quality of published health research with robust reporting guidelines.^19^ However, it fails to put research participants at the centre of care by omitting information to allow healthcare professionals to act quickly and decisively. For clinical research to be ethical, any potential risks for participants must be carefully considered and mitigated against.^20^ This protocol review suggests that participant risk in relation to trials prematurely terminating is not being considered.

About 19% (45/235) of protocols identifying the possibility of a premature termination (95.9%, 235/245), included that the investigator should contact or inform the participant. It is only appropriate that participants and/or parents are informed of the end of their clinical trial by the team they have established, a relationship with and who are likely to be within their standard clinical care team. It could be assumed that healthcare professionals would not need to be told to inform their participants if the trial has been prematurely terminated as this would be an obvious and necessary step. This assumption may explain why so few of the protocols included this direction; however, there are examples of pharmaceutical companies sponsoring clinical trials announcing premature termination publicly.^21,22^ This resulted in participants, families, and healthcare professionals learning the news simultaneously. Trial results affect sponsor’s stock value,^23^ which may explain the decision to control the information publication; however, this removes the opportunity for healthcare professionals to plan future care and request information from the trial team before participants seek information. This creates a potentially challenging and vulnerable environment for healthcare professionals with clinical expertise but who do not have the information to provide adequate support, answer questions or devise a care plan.

After learning that a trial has terminated prematurely, the logical next question for participants, parents, and healthcare professionals would be ‘what now?’. Only 42% (98/235) of the protocols identifying the possibility of a premature termination (95.9%, 235/245), provided information for healthcare professionals relating to participant care, with variation in the quantity and quality of that information. Protocol’s lacking this information will result in delays for participants and, in the case of paediatric participants their parents, while healthcare professionals wait for study sponsors to provide further details. Delays in being able to communicate what the trial termination means for the individual participant and what next steps are required, will be a factor in how all those involved experience this event. Depending on the reason for premature termination, communication delays may have patient safety implications, for example, if a participant continues to take a trial medication. Recognising that trust is a key component in the relationship between patients and healthcare professionals,^24,25^ not knowing the required next steps risks families losing trust in their clinical team and affecting the therapeutic relationship.

Participants and families may need to come back to the research site for examinations, procedures, and to return unused medications. Within this review, protocols detailed that participants should be seen (6.9%, 17/245), the conduct of a specific visit described in the protocol (21.6%, 53/245), additional visits beyond those in the protocol if necessary for safety monitoring (0.8%, 2/245), and any additional procedures the investigator is informed of (6.9%, 17/245). Particularly in early phase trials or trials being delivered by specialists, the research site may be hundreds of miles from the family home and require a parent to make such arrangements as travel, time away from paid employment, and sibling childcare. These practical issues contribute to the burden experienced when participating in clinical research^26^ and are more challenging at short notice.

How the situation is experienced may depend on why participants chose to enrol initially, the other treatment options available, why the trial is prematurely terminated, and the way it is communicated. Premature termination for futility has only been shown to be experienced negatively with a loss of engagement, feeling distressed, being unprepared,^27^ frustration after learning of the discontinuation via the media and being fearful while waiting to be unblinded to their treatment.^28^ Healthcare professionals having the required information to support and clinically manage participants will feed into this experience.

It is understood that providing specific guidance in advance is challenging as at the time of writing the protocol, the reason for premature termination is unknown. If terminating for safety reasons, procedures such as blood tests may be required specifically related to the newly identified risk. However, all trials could provide the initial steps to be taken, acknowledging that additional procedures may be required, and details will be provided by the sponsor. This could be within the protocol itself or a specific document such as a standard operating procedure referenced in the protocol. To further reduce delays and provide healthcare professionals with the most useful information for participant management, sponsors could consider including guidance based on termination categories such as efficacy, inferiority, and futility. The urgency of stopping the trial treatment and commencing an alternative could be very different depending on the reason for termination. Inferiority guidance, where the trial treatment is proving less effective than the control could highlight the need to stop and switch to standard of care or a comparator as soon as possible. It is not possible for healthcare professionals to communicate the news of the trial prematurely terminating in isolation, participants and/or parents will naturally want to know what that means for them and what happens next. Healthcare professionals need sufficient information to have that conversation.

### Limitations

This review only included those trials registered on the clinicaltrials.gov database, this relies on researchers applying to the database for their clinical trial to be registered. Although in the United Kingdom registration to a publicly accessible database is a condition of the Research Ethics Committee’s favourable opinion for clinical trials,^11^ other databases could be used. In addition, trials without available clinical protocols were excluded. Therefore, not all investigational medicinal product trials being delivered in the United Kingdom to children and young people were included, and it is likely that commercially sensitive clinical trials were less represented.

This review only examined the clinical trial protocol and therefore information may have been available to healthcare professionals regarding premature termination and how to manage participants in additional documents or communications.

### Implications for practice

Healthcare professionals delivering research need to be aware of the potential for clinical trials to terminate prematurely and that it may be experienced negatively by both clinical staff, participant’s, and families. Reviewing the protocol during the set-up phase of a trial, specifically for what information is provided for managing participants in this situation would allow healthcare professionals the opportunity to consider in advance how they might manage their participants and families should the situation arise. An awareness of the issue and early protocol review for information in the event of the trial terminating prematurely would allow the development or adaptation of a local guideline.

Those designing clinical trials can improve the experience for healthcare professionals and research participants by including clear directions within the trial protocol. This could be standardised by updating the guidance on which items should be included in clinical trial protocols, such as SPIRIT.^18^

### Implications for research

This review shows that healthcare professionals are unable to depend on the clinical trial protocol including information on participant management and care in the event of a premature termination. There is no published guidance, outside of clinical trial protocols, for how healthcare professionals delivering clinical research can support participants and their families in this situation. It is not known if other documents are being used in practice or if these meet the needs of healthcare professionals and research participants.

## Conclusion

The clinical trial protocol is the key document describing how the trial is to be conducted. This review concludes that while the potential for premature termination is acknowledged in most protocols, there is a lack of information for how healthcare professionals delivering paediatric clinical trials manage and care for participants should this event occur. Healthcare professionals should be aware of the potential for premature termination, understand that clinical trial protocols will not always have clear instructions for this situation and consider how they would manage their trial participants. Researchers writing clinical trial protocols can go further than meeting the regulatory requirements, and consider in the case of the trial prematurely terminating how the participants should be managed by the healthcare professionals delivering the trial.

## Supplemental Material

sj-docx-1-ctj-10.1177_17407745241296864 – Supplemental material for UK paediatric clinical trial protocols: A review of guidance for participant management and care in the event of premature termination Supplemental material, sj-docx-1-ctj-10.1177_17407745241296864 for UK paediatric clinical trial protocols: A review of guidance for participant management and care in the event of premature termination by Helen Pluess-Hall, Paula Smith and Julie Menzies in Clinical Trials
