# Supplementary material for: UK paediatric clinical trial protocols: A review of guidance for participant management and care in the event of premature termination
Source: Clin Trials. 2024 Nov 19;22(2):220–6. doi: 10.1177/17407745241296864 (PMC11986081; doi:10.1177/17407745241296864)
Supplement: sj-docx-1-ctj-10.1177_17407745241296864 – Supplemental material for UK paediatric clinical trial protocols: A review of guidance for participant management and care in the event of premature termination [file sj-docx-1-ctj-10.1177_17407745241296864.docx]

**Supplementary Material**

Supplementary Table 1 - Reasons included within protocols for premature trial termination.

| **Reason** | **Number of Protocols** | **Example** |
| --- | --- | --- |
| Participant safety | 91 | *“…if new toxicological or pharmacological findings or serious adverse events (SAEs) invalidate the earlier positive benefit-risk-assessment.”*  Protocol 16 |
| Participant recruitment | 48 | *“…if the subject recruitment is so slow that the clinical trial cannot be completed within a reasonable time frame.”*  Protocol 3 |
| Non-compliance | 38 | *“…noncompliance…”*  Protocol 85 |
| Revision of the drug development plan | 33 | *“A decision by the Sponsor to suspend or discontinue testing, evaluation, or development of the product.”*  Protocol 216 |
| Data | 24 | *“…lack of evaluable and/or complete data…”*  Protocol 29 |
| Regulatory decision | 22 | *“…decision by FDA or regulators…”*  Protocol 29 |
| Ethical issues | 9 | *“…safety or ethical issues…”*  Protocol 18 |
| Medical reasons | 9 | *“…reasonable medical or administrative reasons.”*  Protocol 53 |
| Administrative reasons | 8 | *“…administrative reasons…”*  Protocol 57 |
| Study will not meet the objective(s) | 3 | *“…if information emerges which demonstrates that the study will not meet its primary objective…”*  Protocol 24 |
| Efficacy | 5 | *“Demonstration of efficacy that would warrant stopping…”*  Protocol 197 |
| Futility | 4 | *“…futility…”*  Protocol 106 |
| Scientific reasons | 1 | *“…scientific or administrative reasons…”*  Protocol 97 |
| Feasibility | 1 | *“Conduct of the study is no longer feasible due to changes in therapeutic landscape…”*  *Protocol 238* |
